# Correlation between circulating tumour DNA and metabolic tumour burden in metastatic melanoma patients

**DOI:** 10.1186/s12885-018-4637-6

**Published:** 2018-07-09

**Authors:** Ashleigh C. McEvoy, Lydia Warburton, Zeyad Al-Ogaili, Liesl Celliers, Leslie Calapre, Michelle R. Pereira, Muhammad A. Khattak, Tarek M. Meniawy, Michael Millward, Melanie Ziman, Elin S. Gray

**Affiliations:** 1 School of Medical and Health Sciences, Edith Cowan University, 270 Joondalup Drive, Joondalup, WA 6027 Australia; 2 Department of Medical Oncology, Sir Charles Gairdner Hospital, Hospital Avenue, Nedlands, WA 6009 Australia; 3 Department of Molecular Imaging and Therapy Service, Fiona Stanley Hospital, Murdoch, WA 6150 Australia; 4 Department of Medical Oncology, Fiona Stanley Hospital, 11 Robin Warren Drive, Murdoch, WA 6150 Australia; 5 School of Medicine and Pharmacology, The University of Western Australia, 35 Stirling Highway, Crawley, WA 6009 Australia; 6 School of Biomedical Sciences, University of Western Australia, 35 Stirling Highway, Crawley, WA 6009 Australia; 7 Centre for Opthalmology and Visual Science, University of Western Australia, 35 Stirling Highway, Crawley, Western Australia 6009 Australia

**Keywords:** Circulating tumour DNA, ctDNA, Metabolic tumour burden, Tumour lesion glycolysis, Melanoma, Droplet digital PCR

## Abstract

**Background:**

Circulating tumour DNA (ctDNA) may serve as a measure of tumour burden and a useful tool for non-invasive monitoring of cancer. However, ctDNA is not always detectable in patients at time of diagnosis of metastatic disease. Therefore, there is a need to understand the correlation between ctDNA levels and the patients’ overall metabolic tumour burden (MTB).

**Methods:**

Thirty-two treatment naïve metastatic melanoma patients were included in the study. MTB and metabolic tumour volume (MTV) was measured by 18F-fluoro-D-glucose positron emission tomography/computed tomography (FDG PET/CT). Plasma ctDNA was quantified using droplet digital PCR (ddPCR).

**Results:**

CtDNA was detected in 23 of 32 patients. Overall, a significant correlation was observed between ctDNA levels and MTB (*p* < 0.001). CtDNA was not detectable in patients with an MTB of ≤10, defining this value as the lower limit of tumour burden that can be detected through ctDNA analysis by ddPCR.

**Conclusions:**

We showed that ctDNA levels measured by ddPCR correlate with MTB in treatment naïve metastatic melanoma patients and observed a limit in tumour size for which ctDNA cannot be detected in blood. Nevertheless, our findings support the use of ctDNA as a non-invasive complementary modality to functional imaging for monitoring tumour burden.

**Electronic supplementary material:**

The online version of this article (10.1186/s12885-018-4637-6) contains supplementary material, which is available to authorized users.

## Background

Melanoma is an aggressive form of skin cancer that is increasing in prevalence worldwide [1]. Metastatic melanoma is a highly aggressive and difficult to treat cancer, particularly when patients present with advanced-stage disease that is unresectable [2]. Recent advances in understanding the molecular mechanisms of melanoma oncogenesis and immune evasion have resulted in the introduction of BRAF and immune checkpoint inhibiting agents. These new treatments have improved the melanoma survival [3–6], with the greatest treatment benefit observed when treatment is initiated at a lower disease burden [2, 7–9].

In patients with advanced stage melanoma, treatment decisions are based upon clinical and imaging findings. In recent years, positron emission tomography with 2-deoxy-2[fluorine-18] fluoro-D-glucose integrated with computed tomography (FDG PET/CT) has emerged as a powerful imaging tool for initial staging and evaluating treatment response in metastatic melanoma [10, 11]. ^18^F–FDG is a radio labelled glucose analogue which reflects tumour metabolic activity. Commonly, FDG PET/CT is used to determine tumour burden as it provides a high tumour-to-background intensity ratio which facilitates computer generated measurements of total body metabolic tumour volume (MTV) and total lesion glycolysis (TLG) from which metabolic tumour burden (MTB) can be quantitatively calculated [12, 13].

As a blood-based biomarker, circulating tumour DNA (ctDNA) offers a non-invasive and easily accessible method of providing a real-time “snap shot” of tumour burden. The level of ctDNA sensitivity however differs between tumour types, AJCC stages, mutant forms and between patients [14]. In AJCC stage IV melanoma patients, ctDNA has been recognised as a valuable biomarker for tumour genetic profiling, monitoring disease progression, response to therapy and as a predictor of clinical outcome [15–23]. Whilst, ctDNA has been detected in 73–89% of patients prior to therapy initiation [15, 16, 19, 24] and the absence of ctDNA in these patients has been suggested as a prognostic marker for a better disease outcome [19], the level of overall tumour burden at which ctDNA can be detected has not yet been quantified.

Numerous platforms are available for the detection of ctDNA, however the droplet digital PCR (ddPCR) platform has been shown to be the most sensitive, capable of detecting mutant DNA (such as *BRAF* V600E) at 0.001% frequency abundance [25, 26]. The lower limit in tumour size that shed detectable amounts of ctDNA into the blood is however unclear, and it may vary between cancer types. This information is critical for clinical validation of the efficacy ctDNA as a non-invasive complimentary method to functional imaging for monitoring of tumour burden in melanoma patients.

Here we determined the levels of ctDNA measured by ddPCR targeting tumour specific mutations in a cohort of 32 treatment naive stage IV melanoma patients. We evaluated whether the presence of ctDNA was associated with progression free survival and whether ctDNA levels correlated with MTB measured by FDG PET/CT.

## Methods

### Patient and sample collection

We retrospectively selected 32 from a cohort of American Joint Committee on Cancer (AJCC) 7th Edition, stage IV melanoma patients enrolled in a multi-centre study from February 2013 to May 2017. Inclusion criteria incorporated patients whose plasma sample was collected within 8 weeks from an FDG PET/CT scan and before any systemic therapy (See Additional file 1 for a detailed list of timing from FDG PET/CT scan to collection of bloods). As part of the patients’ routine diagnostic work out, *BRAF* mutation testing were completed prior to enrolment in the study. Additional mutational profiling for those patients that were considered *BRAF* wild-type was conducted on tumour tissue using Next Generation Sequencing (NGS). Written informed consent was obtained from all patients under approved Human Research Ethics Committee protocols from Edith Cowan University (No. 11543) and Sir Charles Gardner Hospital (No. 2013–246), Western Australia.

### ^18^F-labeled fluorodeoxyglucose positron emission tomography/computed tomography

FDG PET/CT scans were performed on combined PET/CT scanners at approved nuclear medicine centres in Perth, Western Australia. After a minimum fasting period of 6 h, patients were injected with 5 MBq pr. kg ±10% of ^18^FDG (minimum 200 MBq and maximum 600 MBq). Positron emission tomography was performed on patients with serum glucose levels below 11 nmol/l at an acquisition time of 3 min per bed position. To determine anatomical location and for attenuation correction purposes, a whole-body low-dose computed tomography scan was performed. Details of the imaging cameras are outlined (see Additional file 2). After being used for routine clinical purposes, all images were reviewed retrospectively and independently by two experienced nuclear medicine physicians, blinded to the ctDNA analysis. The MTV is calculated from the maximum length of the lesion using CT images obtained from the FDG PET/CT imaging data and the TLG is calculated as MTV x mean SUV in the volume [12]. Analysis was conducted on a Siemens Syngo workstation (Siemens Healthcare GMbH) with each specialist independently reporting the global TLG (MTB) and the global MTV in cm^3^, as per Additional file 2. An average of the two scores provided by the nuclear medicine physicians was used for final analysis.

### Plasma sample preparation and DNA extraction

Blood samples were collected from stage IV melanoma patients, prior to initiation of any systemic therapy, into EDTA vacutainer tubes and stored at 4 °C. Plasma was separated within 24 h by centrifugation at 1600 g for 10 min, followed by a second centrifugation at 2000 g for 10 min, and then stored at -80 °C until extraction. Cell free DNA (cfDNA) was isolated from between 1 to 5 mL of plasma using the QIAamp Circulating Nucleic Acid Kit (Qiagen) as per the manufacturer’s instructions and eluted in 40 μl AVE buffer (Qiagen) and stored at -80 °C until ctDNA quantification.

### CtDNA quantification

CtDNA was quantified by ddPCR as previously described [16, 27]. Briefly, a PCR mixture containing 1 x ddPCR supermix, primer/probe sets for *BRAF* [see Additional file 2] (custom synthesized by Life Technologies), *NRAS* (Bio-Rad) or *KIT* (Bio-Rad) and 5 or 8 uL cfDNA were emulsified with droplet generation using the Automatic Droplet Generator (AutoDG, Bio-Rad). Amplifications were performed using cycling conditions previously described [16]. Droplets were analysed through a QX200 droplet reader (Bio-Rad) and data was analysed using QuantaSoft analysis software (Bio-Rad). To ensure the accuracy of results, each sample was tested minimally in duplicate. For quantification, a minimum of 10,000 acceptable droplets per 20 uL reaction was required.

### Statistical analysis

Association between patient characteristics and ctDNA positivity or negativity was calculated by Fisher’s exact tests or unpaired T-test where appropriate. To assess the inter-observer agreement, Pearson’s correlation analysis was performed and an evaluation of bias and precision was performed using Bland Altman analysis [28]. The correlation between MTV and MTB and the number of mutated copies of ctDNA was evaluated using Pearson’s correlation after log-transformation of both variables. Progression free survival (PFS) was calculated as the time from the date of initiating therapy to the date of first reported progressive disease (PD) or censored with stable disease at the most recent visit. Median PFS was calculated using the Kaplan-Meier method and compared using the log-rank test. Cox proportional hazards regression analyses were performed to examine association of ctDNA, and other variables with PFS. Multivariate Cox regression models were evaluated using a stepwise approach with bidirectional elimination to determine the best fit model. Confidence intervals were set at 0.05 and significance levels are reported as *P* < 0.05. Statistical analyses were performed using Statistical Package for Social Sciences for Window version 22 (SPSS, Chicago, IL) and plotted using GraphPad Prism version 5.

## Results

### Cohort

A total of 32 patients with stage IV metastatic cutaneous melanoma were included in the study. Patient characteristics are shown in Table 1. The median time between blood collection and PET/CT was 1.9 weeks (range: 0.1–8 weeks, see Additional file 1). Patients were treated with targeted therapy and/or immunotherapy (see Additional file 1). After a median follow-up of 64.4 weeks (95% CI: 46.2–82.6 weeks), 11 patients progressed whereas a large proportion retained control of the disease.

**Table 1:** Patient characteristics

	Total
	(*n* = 32)
Gender	
Male	62.5 (20)
Female	37.5 (12)
Age	
Median years (range)	57 (25–83)
Stage	
M1a	12.5 (4)
M1b	6.3 (2)
M1c	81.3 (26)
Performance status, ECOG	
0	65.6 (21)
1- 2	34.4 (11)
LDH	
Not elevated	34.4 (11)
Elevated	21.9 (7)
Not available	43.8 (14)

*Abbreviation*: *ECOG* Eastern Cooperative Oncology Group, *LDH* Lactate dehydrogenase. * Significant difference *p* < 0.05

### Evaluation of plasma ctDNA

**Fig. 1:**
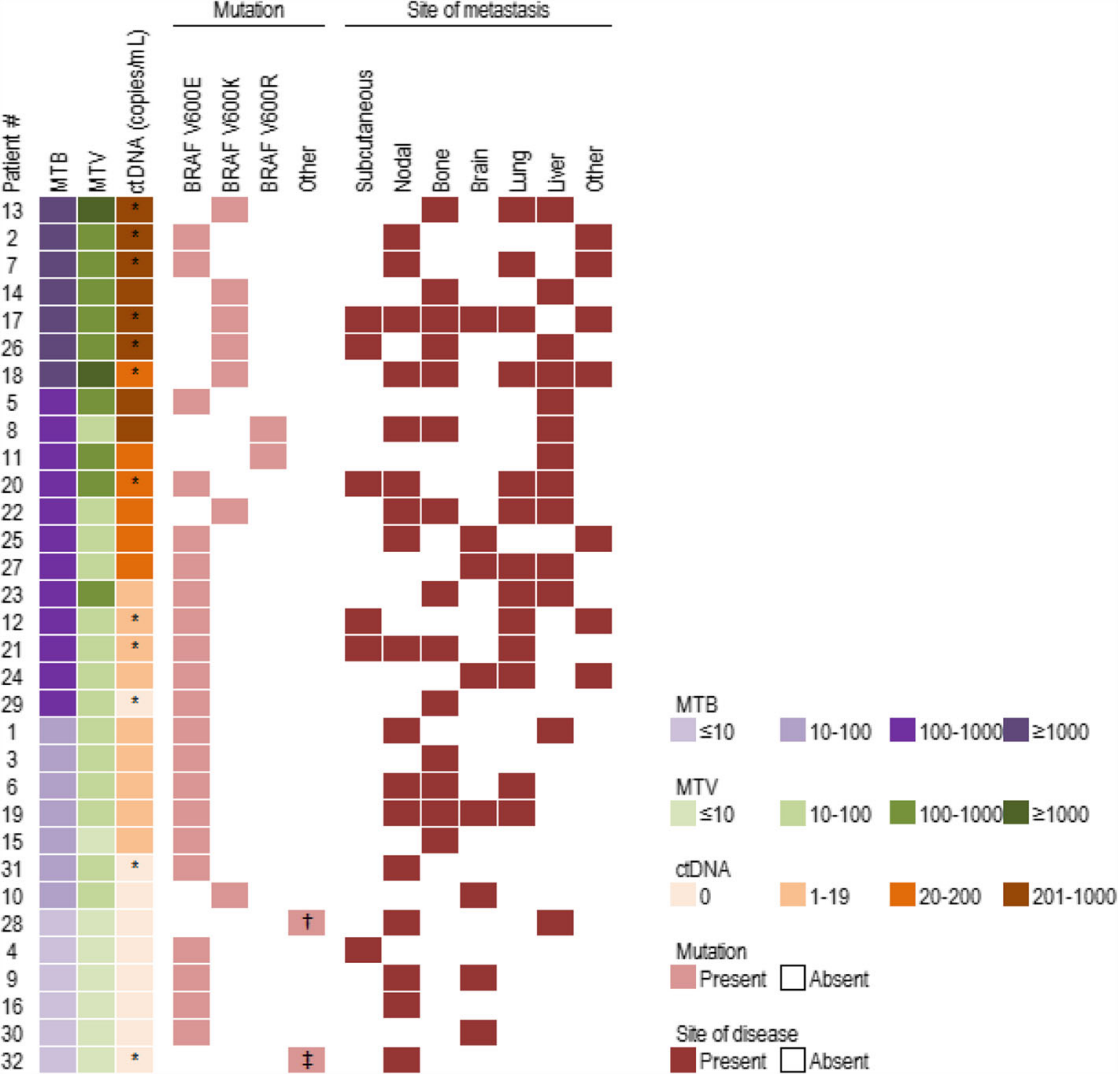
Overview of disease burden and ctDNA for 32 patients with melanoma. Patients are ordered according to metabolic tumour burden (MTB). Other indications of tumour burden such as metabolic tumour volume (MTV) and site of metastasis are compared with circulating tumour DNA (ctDNA, copies/mL of plasma) levels and site of metastasis. CtDNA was measured by droplet digital PCR targeting the mutations indicated. *denotes cases for which cfDNA was extracted from 1 mL of plasma. †denotes *KIT* L576P, ‡denotes *NRAS* Q61L

**Fig. 2:**
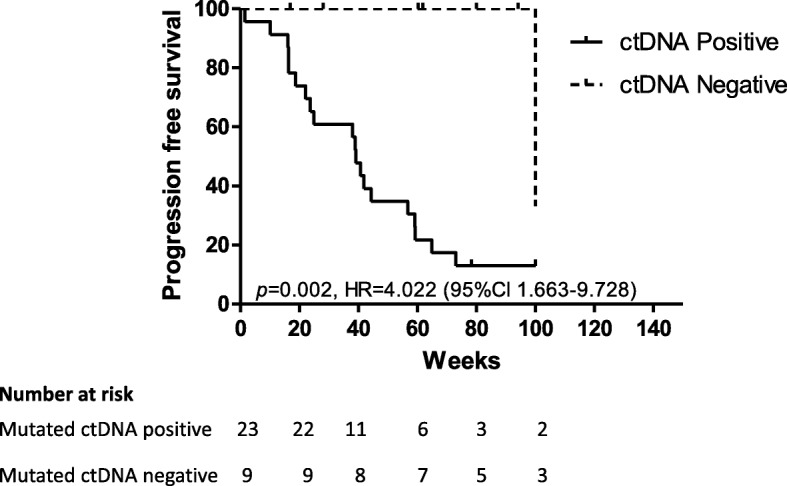
Kaplan-Meier curve for progression free survival (PFS) according to the presence of circulating tumour DNA (ctDNA) in plasma. In patients with detectable ctDNA, a median PFS of 33.29 weeks was observed

Of the 32 tumour samples analysed, at least one mutation was known to be present with 94% of the samples harbouring a mutation in *BRAF* V600. Other mutations detected in ctDNA included *KIT* L576P (patient 28) and *NRAS* Q61L (patient 32) (Fig. 1). CtDNA was detected in 23 patients with a median concentration of 38 copies/mL of plasma (range: 1.6–52,440 copies/mL). The remaining 9 patients had no detectable ctDNA. A comparison of patient characteristics was conducted between the ctDNA positive and ctDNA negative patients (Table 2). Significant differences were evident in sex (*p* = 0.05), with a higher proportion of males having detectable ctDNA than females, and M stage (*p* = 0.03), with M1c (patients with visceral metastases) more likely to have detectable ctDNA than M1a and M1b. When evaluating for PFS, patients with detectable ctDNA had a significantly shorter median PFS (39.14 weeks) than patients with undetectable ctDNA (102.29 weeks, *P* = 0.002; Fig. 2).

**Table 2:** Comparisons of clinical characteristics between patients with and without ctDNA present (*N* = 32)

	ctDNA+	ctDNA-	*P* value
	(*n* = 23)	(*n* = 9)	
Gender			
Male	85.0 (17)	15.0 (3)^*^	0.05^*^
Female	50.0 (6)	50.0 (6)	
Age			
Median years (range)	58 (25–78)	62 (34–83)	0.24
Stage			
M1a or b	16.7 (1)	83.3 (5)	0.03^*^
M1c	84.6 (22)	15.4 (4)	
Performance status, ECOG			
0	69.6 (16)	30.4 (5)	0.68
> 0.99	63.6 (7)	36.4 (4)	
LDH			
Not elevated	63.6 (7)	36.4 (4)	0.60
Elevated	85.7 (6)	14.3 (1)	
Not available	71.4 (10)	28.6 (4)	

*Abbreviation*: *ECOG* Eastern Cooperative Oncology Group, *LDH* Lactate dehydrogenase. ^*^Significant difference *p* < 0.05

### Inter-observer analysis of MTV and MTB

MTV and MTB were assessable in all patients from PET/CT scans. A good correlation (R^2^ =. 0.978 and 0.998 respectively) was observed between the two independent scores (see Additional file 3), with Bland-Altman plots showing a good agreement between the two specialists, with all but one observation falling within 95% CI.

### Correlation between ctDNA copies / mL and MTV/MTB

**Fig. 3:**
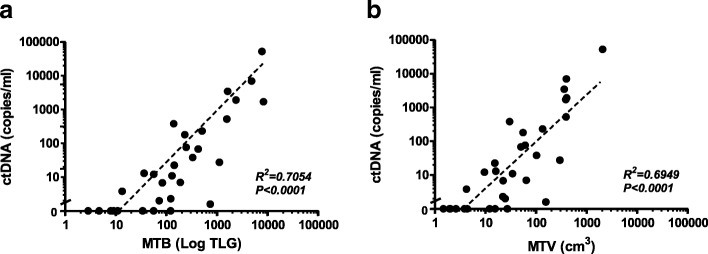
Correlation between circulating tumour DNA (ctDNA) levels and disease burden. Correlation between ctDNA levels in copies/mL of plasma and MTB (**a**) or MTV (**b**) assessed by FDG-PET imaging. Analysis was performed by using Spearman rank correlation

A significant correlation (R^2^ = 0.7054, *P* < 0.0001) (Fig. 3a) was found between the copies of ctDNA per mL of plasma and the global TLG or MTB. Similarly, a weaker but none the less significant correlation was observed (R^2^ = 0.6949, *P* < 0.0001) (Fig. 3b) between the copies of ctDNA per mL of plasma and the MTV. CtDNA was undetectable in all patients with an MTB value of ≤10, which suggests that this is the lower limit of disease burden at which ctDNA can be detected. CtDNA was also undetectable in three cases with MTB > 10, namely patients 10, 29 and 31 (Fig. 1). CfDNA was isolated from 1 mL of plasma for patients 29 and 31 and patient 10 had a cerebellar lesion only brain metastasis which may reduce our capacity to detect ctDNA in these cases.

### Multivariate cox regression analyses of PFS

Finally, in a univariate Cox regression analysis, detectable ctDNA, high MTB and M1c disease were found to be significantly associated with decreased PFS (Table 3). A multivariate Cox regression analysis revealed that these three variables do not independently predict PFS. Using a stepwise approach with bidirectional elimination to determine the best fit model, we found that ctDNA explains most of the differences in PFS with an adjusted HR = 7.658 (CI 95% 1.762–33.283) (Table 3) after controlling for sex, age and ECOG status.

**Table 3:** Factors associated with PFS

Factor	Variable	Univariate analysis			Multivariate analysis		
		*p*-value	HR	CI (95%)	p-value	HR	CI (95%)
ctDNA	positive/negative	0.009^*^	6.973	1.612–30.165	0.007^*^	7.658	1.762–33.283
Age	Continuous	0.619	0.992	0.961–1.024	0.558	0.988	0.949–1.029
Sex	Male vs. Female	0.419	0.834	0.537–1.295	0.548	1.184	0.682–2.058
ECOG	0–2	0.747	1.127	0.544–2.337	0.526	1.284	0.593–2.781
MTB$	> 34 vs. ≤34	0.018^*^	5.845	1.356–25.206			
Stage	M1a,b vs. M1c	0.035^*^	1.454	0.218–0.945			

*Abbreviations*: *ctDNA* circulating tumour DNA, *ECOG* Eastern Cooperative Oncology Group, *MTB* Metabolic Tumour Burden, *HR* hazard ratio, *CI* confidence interval. *Statistically significant. $Dichotomised at First Interquartile

## Discussion

Here we demonstrated a significant correlation between the ctDNA levels in plasma and MTB measured by PET/CT in metastatic melanoma patients. Moreover, we observed a threshold for detecting ctDNA at an MTB score of ≤10. Finally, we confirm previous reports showing that undetectable ctDNA is associated with longer PFS.

With regards to patients with undetectable ctDNA, several cases are worth highlighting. In our study, one case (patient 4) with subcutaneous metastases only and noted to have low disease burden, was found negative for plasma ctDNA. Previously, subcutaneous metastases have been shown to be associated with low levels of ctDNA, despite extensive disease [22]. Patients 16, 31 and 32 had nodal metastases only and ctDNA was not detectable. Whilst Wong et al. [22] have shown that patients with nodal involvement often display high levels of ctDNA, we did not confirm this finding. It is worth noting that no other patients in our cohort had nodal disease alone, thus extrapolation of our results suggesting that ctDNA is undetectable in node only disease should be considered with caution. Moreover, this may be confounded by the fact that in two cases (patients 16 and 32) the MTB was below our threshold of 10. The third patient (patient 31) had an MTB of 33.49 but the lack of ctDNA could be attributed to the fact that cfDNA was extracted from only 1 ml of plasma.

Undetectable ctDNA in patients 9, 10 and 30 may be explained by the sites of their metastatic disease, nodal and brain (patient 9) and brain only (patient 10 and 30). Previously, low or undetectable ctDNA levels have been observed in patients with brain metastases [22, 29, 30].

One significant outlier is patient 29, where despite having bone metastases and a significant MTB, we were unable to detect ctDNA. Bone metastases have previously been associated with high levels of ctDNA [22]. In this case, cfDNA was also extracted from only 1 mL of plasma which is likely to have a significant impact on detection levels of ctDNA [31].

Remarkably patient 28 (with nodal and liver metastases) had undetectable ctDNA however, MTB was ≤10. No obvious limitations were evident in this case, particularly with regards to the volume of plasma collected. Thus, we are confident that with our current assay sensitivities, we are not able to detect ctDNA in patients with a MTB value of ≤10, regardless of disease site or mutation. Nevertheless, this observation needs to be corroborated in larger studies, taking in consideration diverse mutated genes and the site of metastases.

To our knowledge, this is the first study in melanoma that has directly compared the level of ctDNA with the exact MTB calculated from the sum of TLG for all evaluable lesions. Recently, Wong et al. [22] showed that ctDNA levels correlate with qualitative analysis of whole body MTV in metastatic melanoma patients. Similar to our findings, a strong correlation was observed between ctDNA levels and MTV (*r* = 0.61; *P* < 0.001). Whilst it is difficult to make a direct comparison between this study and our own due to the different methodologies employed to detect ctDNA (digital PCR and targeted sequencing) and different reporting mechanisms of MTV (Wong reported results in mL, whilst we reported results in cm^3^), it is interesting to note there is a remarkable difference in the median levels of plasma ctDNA reported by both studies. Wong et al. reported a median ctDNA concentration of 1112 copies/mL of plasma (range 63–97,000) which is considerably higher than the copies/mL of plasma that we reported, indicating an enrichment of patients with lower disease burden in our study. Finally, our measurements incorporated PET parameter TLG to define all measureable lesions combined with the metabolic activity of each tumour, thus measuring both tumour volume and aggressiveness. This approach provides a comprehensive score of not only of tumour burden but also of tumour activity and therefore, an overall perspective of the disease status of patient.

In line with our study, the presence of ctDNA has been directly correlated with patient survival and MTB in advanced stage non-small cell lung cancer (NSCLC) [12]. This study assessed allele frequency measured by NGS in 24 NSCLC patients and MTB (calculated from the sum of TLG for all evaluable lesions) and found a significant correlation (*P* = 0.001). The authors also reported a significantly shorter median overall survival (OS) in patients with detectable ctDNA compared to those with no detectable ctDNA. In our study, we have specifically chosen not to assess OS as this endpoint includes death from any cause, and thus is influenced by co-morbidities and access to systemic therapies over different periods of time. For this reason, our analysis focused on PFS, which was significantly shortened in patients with detectable ctDNA. Finally, multivariate Cox regression analyses demonstrated that ctDNA is not an independent variable but rather a reflection of other disease burden measurements such as MTB and disease stage.

There are a number of noteworthy limitations to our study, largely associated with its retrospective nature. Firstly, the timing of blood collections in accordance with PET/CT imaging was varied. In the Winther-Larsen and colleagues study [12], blood samples were collected at the time of inclusion into the study, and PET/CT imaging. This resulted in a median interval of 2 days between imaging and blood draw, which is considerably shorter than our median of 1.9 weeks. However, for both studies the timing between blood collection and imaging may be an important factor to consider. Given that ctDNA has a half-life of less than 2 h and have been shown to increase as new lesions become apparent [32], ideally blood draw should be conducted immediately after imaging to ensure that ctDNA detected is a true reflection of lesions identified in the image. Importantly, in all our cases, plasma was collected after imaging and prior to treatment. Secondly, PET/CT imaging was conducted at different institutions, which may have resulted in inter-institutional differences in quality control and scanning [33]; the use of multiple scanner models has been associated with variability in the standard uptake of FDG readings [34]. We did not however observe any substantial deviation in the correlation between ctDNA and MTB due to scanner models (see Additional file 4). Finally, we acknowledge that this cohort is heavily biased for *BRAF* V600 mutated cases, and future studies should address this across multiple mutations.

## Conclusions

In conclusion, the significant correlation of ctDNA with MTB in treatment naïve metastatic melanoma patients in this study suggests that quantification of ctDNA between scans may provide a minimally invasive option with which to detect changes in disease burden in melanoma. We observed a limit in tumour size for which ctDNA can be detected in blood, suggesting that detection of ctDNA in patients with low disease burden necessitates further improvements in the technology to increase sensitivity.

## Additional files

Additional file 1: Detailed list of timing from FDG PET/CT scan to collection of bloods and systemic treatments that patients received after FDG PET/CT and bloods. (XLSX 10 kb)

Additional file 2: Additional methodologies. (DOCX 16 kb)

Additional file 3: Concordance between nuclear physician MTB and MTV measurements. Correlations between the two analysts for MTB (A) and MTV (C). Bland-Altman plot for analysis of agreement for MTB (B) and MTV (D). (PPTX 64 kb)

Additional file 4: Correlation between MTB and ctDNA labelled according to the different scanner types. (PPTX 53 kb)

## Abbreviations

AJCC: American Joint Committee on Cancer
cfDNA: Cell free DNA
ctDNA: Circulating tumour DNA
ddPCR: Droplet digital PCR
FDG PET/CT: 18F-fluoro-D-glucose positron emission tomography/computed tomography
MTB: Metabolic tumour burden
MTV: Metabolic tumour volume
NGS: Next Generation Sequencing
NSCLC: Non small cell lung cancer
OS: Overall survival
PD: Progressive disease
PFS: Progression free survival
TLG: Total lesion glycolysis
