# Supplementary material for: Correlation between circulating tumour DNA and metabolic tumour burden in metastatic melanoma patients
Source: BMC Cancer. 2018 Jul 9;18:726. doi: 10.1186/s12885-018-4637-6 (PMC6038195; doi:10.1186/s12885-018-4637-6)
Supplement: Supplementary file 2 — Additional methodologies. (DOCX 16 kb) [file 12885_2018_4637_MOESM2_ESM.docx]

# Additional file 2

# ^18^F-FDG PET/CT imaging

Patients were required to fast 6 hours before PET/CT examination with glucose level below 11 mmol/L at the time of 18F-FDG injection. 18F-FDG dosage was weight based: weight (kg) _x 4.8 MBq. PET/CT was acquired using dedicated PET/CT scanner (Discovery 710, Biograph 64, 128, 1080) 60 minutes after 18F-FDG injection which provided three-dimensional acquisition, processing and display of CT, PET and PET/CT images, with 4.2 - 6.0 mm PET spatial resolution. First, a low dose CT (30mA, 120 kVp, field of view 500 mm, length of scan 1.0–1.5 m with a speed of 100 mm/sec and a spatial resolution of 1 mm) was performed from the tip of the skull to the level of the proximal thigh or to feet. Then, the acquisition of PET emission images was performed (2–3 minutes per bed position of 8.4 cm). The total acquisition time, accumulating between 100 and 150 million useful events, varied between 25 and 35 minutes per patient. The CT data were used for attenuation correction of PET emission images, and for fusion with PET data for accurate localization of lesions. Non-attenuated data were reconstructed after scan acquisition had been completed. Reconstruction of attenuation corrected data was executed concurrently.

# MTV and MTB assessment

Metabolic tumour volume (MTV) and total lesion glycolysis were semi-automatically calculated using *Syngo Via* VB10b platform. A region of interest (ROI) was drawn around each lesion. The SUV threshold used by the software is 40% of the maximum SUV (SUVmax). When lesion has high SUV (>6) an SUV threshold of 2.5 was used instead. The metabolic tumour burden (MTB) is the sum of all lesions TLG.

BRAF Primers:

5’- CTACTGTTTTCC TTTACTTACTACTACACCTCAGA-3’ (forward) and 5’-ATCCAGACAACTGTTCAAACTGATG-3’ (reverse).

BRAF probes:

T1799-VIC WT (VIC-CTAGCTACAGTGAAATC-MGBNFQ) and A1799-FAM V600E (6FAM-TAGCTACAGAGAAATCMGBNFQ) or AA1798-1799-FAM V600K (6FAMTAGCTACAAAGAAATC-MGBNFQ).

# *BRAF*, *NRAS* and *KIT* cycling conditions:

1 cycle at 95°C for 10 minutes, 40 cycles at 94°C for 30 seconds and 55°C for 1 minute, followed by 1 cycle at 98°C for 10 minutes.
