# Supplementary figures and images for: Correlation between circulating tumour DNA and metabolic tumour burden in metastatic melanoma patients
Source: BMC Cancer. 2018 Jul 9;18:726. doi: 10.1186/s12885-018-4637-6 (PMC6038195; doi:10.1186/s12885-018-4637-6)

## Slide 1
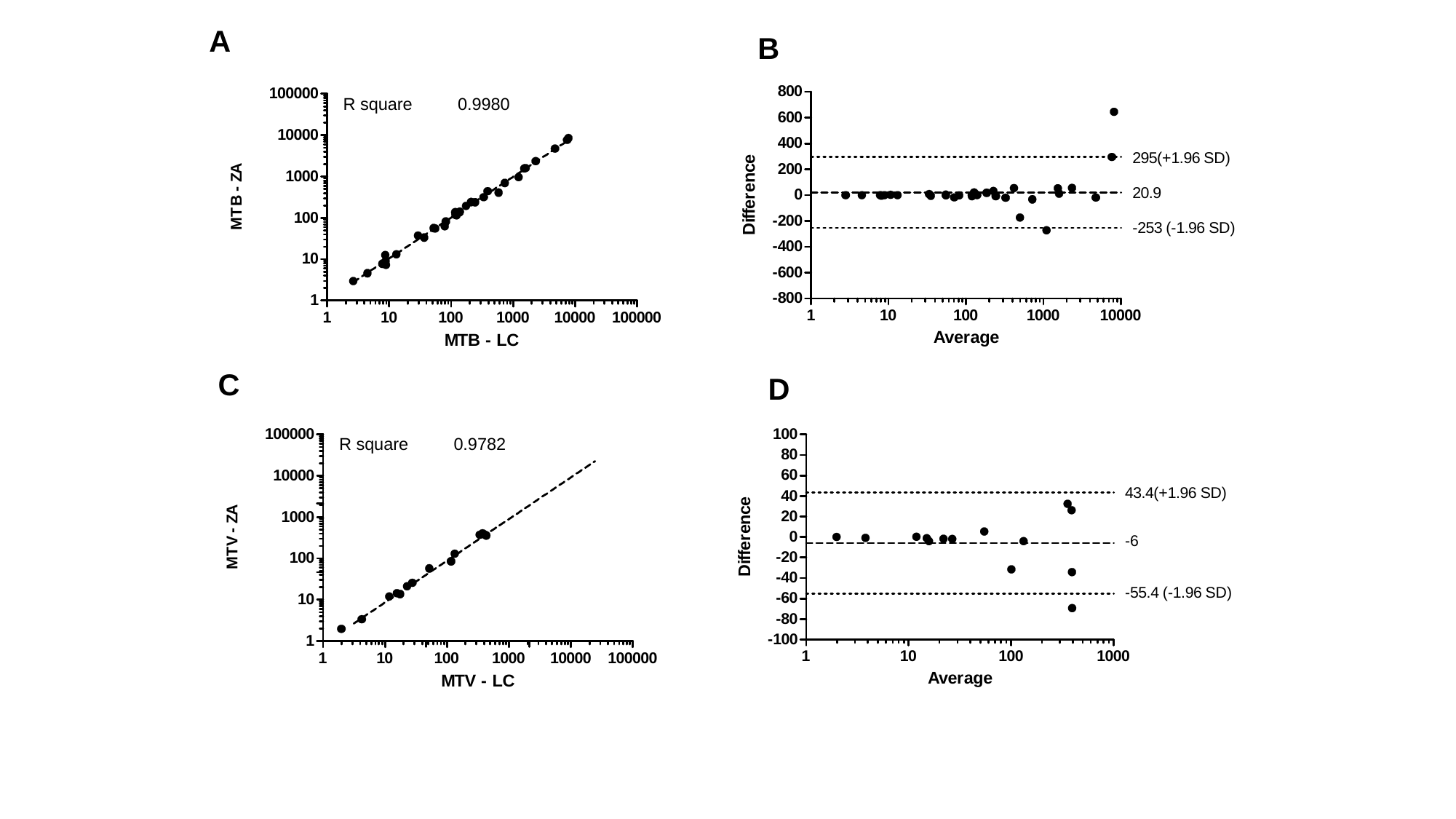

Supplement: Supplementary file 3 — Concordance between nuclear physician MTB and MTV measurements. Correlations between the two analysts for MTB (A) and MTV (C). Bland-Altman plot for analysis of agreement for MTB (B) and MTV (D). (PPTX 64 kb) [file 12885_2018_4637_MOESM3_ESM.pptx]

## Slide 1
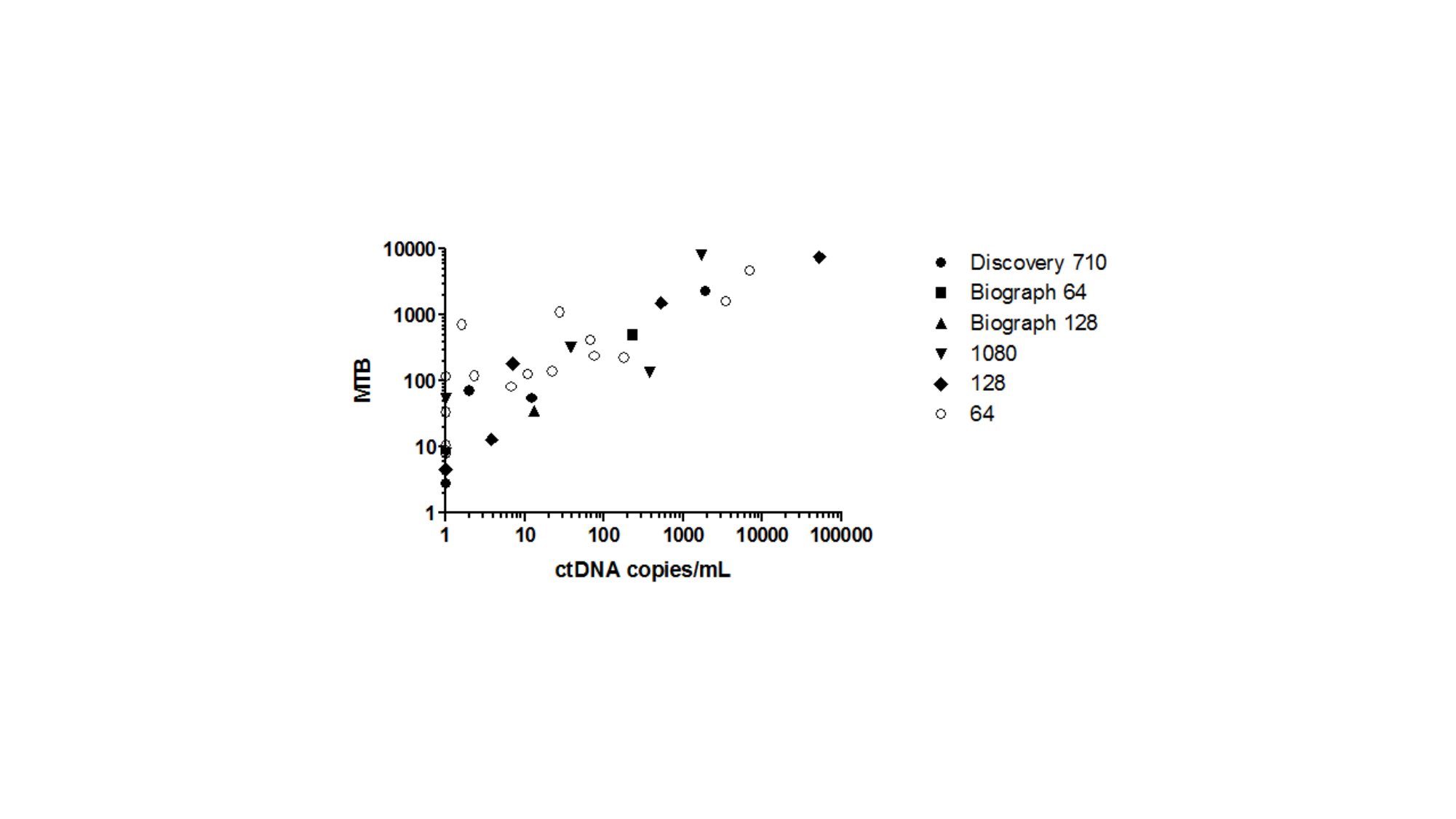

Supplement: Supplementary file 4 — Correlation between MTB and ctDNA labelled according to the different scanner types. (PPTX 53 kb) [file 12885_2018_4637_MOESM4_ESM.pptx]
